# Bacterial Therapy

**Published:** 1912-10-15

**Authors:** W. O. Wetmore

**Affiliations:** New York


### BACTERIAL THERAPY.

BY W. O. WETMORE, M.D., NEW YORK.

Bacterial therapy has appeared as the result of investigations in immunity, especially that conducted by Sir A. E. Wright of St. Mary's Hospital, London, and depends upon the power of dead bacteria in proper doses and menstrum, when injected into an individual, of stimulating the production of antibodies in the tissues, thereby acting as a protecting or curative agent. These resisting elements occur as the result of the presence of bacteria or their toxines in disease, and upon their elaboration recovery depends.

Immunity may be natural or artificial, and is a fortification against disease. The body is fairly well protected from the entrance of pathogenic organisms. The skin is well adapted to the purpose as is the mucous membrane of the alimentary canal, and as are the protecting coats guarding the various avenues of entrance.

Certain substances, probably glandular products, are normally present in the tissues and inhibit or destroy invading bacteria and their toxines. It is reasonably certain, too, that infection is dependent upon the quantity of organisms entering. When infection occurs a reaction is set up which is known as inflammation. The fixed cells proliferate. Certain white cells which have been rolling along the lumen of the blood vessels suddenly migrate by diapedesis or endeavor to capture and destroy by ingestion the free bacteria. They become extremely numerous, too, and should the invading organisms destroy these phagocytes the contained enzymes are liberated and the blood becomes surcharged with these resisting elements. An endeavor is made to wall off the intruders with their poisonous products by the cell elements. An area occurs where the circulation is interfered with and all the elements disintegrate to form pus. Through a process of necrosis this is forced to a surface and discharged. This process is present where pyogenic bacteria are concerned. The walling appears in diseases where local reactions are manifested in most social self limited diseases.

The next effort made by Nature to prevent invasion by disease is through rise of temperature (fever), which is thought to exert a detrimental influence on microbe life.

All this time antibodies are being formed to counteract the particular toxine eliminated by the bacteria. These antibodies may be agglutinins, bacterins, or opsonins, but whichever appears, the production will continue after the organism has disappeared and will be abundant to overcome the invading elements, providing always that sufficient time is allowed for their elaboration. But just here is the trouble; sometimes the toxines are produced in such abundance and are so deadly that sufficient resistance does not occur and death ensues.

Nature, in the process of cure, undertakes to confine the invading organisms at the point of entry or colonization, and the greatest number of fixed cell receptors and detached receptors are here produced. The general system is shut away from the stimulating influences of the bacteria and their products and probably produces no great amount of antitoxine in consequence.

When solutions of dead bacteria in proper quantity are injected into an individual, it would seem that not only locally do the cell elements receive extra stimulation to manufacture antibodies, but the whole system begins the production of receptors against bacterial invasion, while the invading organisms are being checked at the point of invasion. This is shown by a rising opsonic index, which is sometimes remarkable.

This however, does not occur at once, and can be augmented by several increasing doses, given at intervals, depending upon the disease process involved. There is a temporary interval after the administration before the beneficial influence is manifest. The period is apt to be some twelve to twenty-four hours, depending upon the organism, and during this time some discomfort may be felt, but, fortunately, no serious effects have been known to have been produced by their use.

A good illustration of the difference in the therapeutical application of bacterins may be had in compating their use in pneumonia and typhoid fever. In pneumonia a physician is usually summoned at the onset of the disease, because of its sudden manifestation. Several doses are advised at intervals of eighteen hours during the first six days of the disease. This will prepare the system to show a high opsonic index at crisis, and this ordinarily is all that is required to result in recovery. Encouraging hyperemia through injected areas is useful in bringing the curative products to the parts, but should be begun only after one or two doses have been given.

Should one be called late in the disease, say the seventh or eighth day, serum treatment is indicated, because of its immediate effect and because the bacterin, if used, would give a maximum effect only after crisis. Then, too, it might prevent the natural rise which occurs on or about the ninth day. The use of bacterins early and serums late would be still better treatment.

In treating typhoid fever, it is advised to give doses some five days apart, and a general amelioration of symptoms may be expected, beside an early cessation of high temperature. This result is brought about by a fortification of this system with antibodies, and there is not apt to be the condition of autoinfection so frequently seen in this disease.

Serum and bacterial therapy have been confounded at times, especially the terms employed, due largely to the use by Sir A. E. Wright of the term vaccine when using dead typhoid bacteria suspended in salt solution as an immunizing agent against typhoid fever. The term has been continued when using the same product in the treatment of the disease. A better term, it seems to me, for the therapeutic agents would be *bacterins*, as suggested by Doctor Cohen and Doctor Stewart of Philadelphia, leaving the term vaccine to the products used for immunizing purposes, whether by the method of Jenner or Wright.

When an animal is treated in order to produce antitoxine it receives toxine produced by bacterial growth or bacteria themselves in increasing doses, with the result that the blood finally becomes charged with antibodies, and these may be separated from the blood of the animal and transferred to the blood of an individual suffering with the disease. This acts as a curative agent. The only question involved is the use of a sufficient amount to counteract the toxines found in the body of the patient treated.

In using a bacterin an individual so treated is made to manufacture his own antibodies, and while the process is slower, due to the necessity of several injections and the lapse of time between doses, the results are in many instances more gratifying, and the immunity is much more lasting and satisfactory than with serum treatment. It is natural that the serum should disappear within a very few hours, but the antibodies produced by bacterins are withheld over a long period of time.

A certain amount of knowledge of doses is required in using bacterins, but the scale furnished by Sir A. E. Wright is sufficient for ordinary knowledge as a working basis. These figures represent the number of bacteria in one c.c. of normal salt solution:

| | | | |
|---|---|---|---|
| Bacillus coli | 5 | to | 50,000,000 |
| Gonococcus | 5 | to | 50,000,000 |
| Neorformans | 50 | to | 100,000,000 |
| Pneumococcus | 5 | to | 50,000,000 |
| Staphylococcus | 50 | to | 1,000,000,000 |
| Streptococcus | 10 | to | 25,000,000 |
| Typhus bacillus | 5 | to | 50,000,000 |
| Pyocyaneus | 5 | to | 50,000,000 |

The beginner should first use minimum doses. In typhoid it is well to remember that a reaction may occur and is not harmful, but might be annoying to the physician if not anticipated.

The products known as new tuberculin T. R. and B. E. are being used with good effect by those who are able to appreciate that a very minute dose is all that is required to effect a favorable result. The better product would seem to be B. E., which contains the whole cryptoplasmic material prepared by the destruction of the tubercle organism in a ball mill for some six weeks, standardized as to the quantity contained in equal parts of water and glycerin.

It is well to continue at this figure for several doses before increasing and depend for results upon small doses over a long period of time, rather than by a system of progression for a shorter time. This method is unattended by the danger of reaction so often seen in a rapidly increasing plan. The only troublesome cases encountered would be those rare instances where the initial dose was troublesome.

I suggest that an index of susceptibility might be had by using Moro's test and using a serial dilution of this ointment in order to discover any supersensibility to tuberculin products, depending upon the parallelism in reactions for this information.

Altogether it seems that the use of bacterins is founded on a far more rational basis than most other therapy, and only requires the confidence of the user to make it valuable in his hands. Certainly it is but the stimulating of natural processes, which after all should be the main guide in medication.—Reprinted from *New York Medical Journal*, May 4, 1912.

